# *PRSS1*, *SPINK1* Mutations and Associated Factors in Vietnamese Patients With Chronic Pancreatitis

**DOI:** 10.1002/jgh3.70275

**Published:** 2025-09-18

**Authors:** Tran Thi Luong Vo, Hoang Anh Vu, Chuong Quoc Ho, Nguyen Phuoc Ma, Dung Dang Quy Ho, Hoang Huu Bui

**Affiliations:** ^1^ Department of Internal Medicine University of Medicine and Pharmacy at Ho Chi Minh City Ho Chi Minh City Vietnam; ^2^ Department of Gastroenterology Cho Ray Hospital Ho Chi Minh City Vietnam; ^3^ Center for Molecular Biomedicine University of Medicine and Pharmacy at Ho Chi Minh City Ho Chi Minh City Vietnam; ^4^ Department of Endoscopy Cho ray Hospital Ho Chi Minh City Vietnam; ^5^ Department of Gastroenterology University Medical Center at Ho Chi Minh City Ho Chi Minh City Vietnam

**Keywords:** chronic pancreatitis, idiopathic chronic pancreatitis, *PRSS1*, risk factors, *SPINK1*

## Abstract

**Aims:**

Mutations in the *PRSS1* and *SPINK1* genes are recognized as important risk factors for chronic pancreatitis (CP); however, their clinical relevance in Vietnamese populations remains unclear. This cross‐sectional study investigated the prevalence and associated factors of these mutations in Vietnamese CP patients.

**Methods and Results:**

CP was diagnosed according to the 2020 American College of Gastroenterology Clinical Guidelines. Genetic analysis was performed via Sanger DNA sequencing. One hundred sixty CP patients were included from December 2022 to June 2024 at Cho Ray Hospital, Vietnam. Pathogenic mutations were identified in 64 patients (40.0%), with *SPINK1* mutations found in 36.8% and *PRSS1* mutations in 4.4%. The most frequent *SPINK1* variants were c.101A>G (23.7%) and c.194+2T>C (14.3%), and their prevalence was highest in idiopathic CP cases. Multivariate logistic regression analysis revealed that younger age (OR: 0.95; 95% CI: 0.92–0.98), diabetes mellitus (OR: 2.55; 95% CI: 1.11–6.04), pancreatic duct stones (OR: 7.08; 95% CI: 2.81–20.40), and prior surgical intervention (OR: 4.14; 95% CI: 1.34–14.10) were independently associated with pathogenic mutations.

**Conclusion:**

These findings suggest a high prevalence of *SPINK1* mutations, particularly c.101A>G and c.194+2T>C, among Vietnamese CP patients. The significant factors associated with genetic mutations were younger age, diabetes mellitus, pancreatic duct stones, and prior surgical intervention.

**Abbreviations**
ACP: alcoholic chronic pancreatitis
BMI: body mass index
CI: confidence interval
CP: chronic pancreatitis
CT: Computed tomography
DM: diabetes mellitus
ICP: idiopathic chronic pancreatitis
OR: odds ratio
SCP: smoking‐associated chronic pancreatitis

## 1 Introduction

Chronic pancreatitis (CP) is a long‐lasting inflammatory disease leading to irreversible destruction of the pancreatic parenchyma and replacement with strong fibrosis. These permanent morphological changes lead to both exocrine and endocrine dysfunctions, resulting in a significant decline in quality of life, an increased risk of pancreatic cancer, and other complications that shorten life expectancy [1]. Despite substantial advancements in diagnosis and treatment, no currently available therapies can effectively alter the course of the disease, resulting in a poor prognosis. However, the prevalence and incidence of CP have been increasing in recent years in Western countries and the United States [2, 3, 4]. A similar upward trend has also been reported in China, India, Japan, and other Asian countries [5, 6, 7]. Therefore, additional research is needed to investigate the pathogenesis and risk factors for CP to develop more effective interventions aimed at improving disease prognosis.

For many years, alcohol consumption has been considered the main cause of CP. The majority of CP cases in the United States, Japan, and other developed countries are attributed to alcohol consumption [1, 8]. Recently, cigarette smoking has also been indicated to be a significant risk factor for CP [9, 10, 11]. Additionally, in approximately 10%–30% of cases, no identifiable etiology can be identified despite a comprehensive evaluation, which is referred to as idiopathic CP (ICP) [12]. According to recent studies, CP is thought to result from complex gene–environment interactions that may trigger or affect the pathogenesis and progression of the disease [13]. Consequently, the causative role of genetic variants in the development of CP has been recognized, with mutations in the two genes *PRSS1* and *SPINK1* being the most frequently implicated [14, 15]. Pathogenic variants in *PRSS1* stimulate autoactivation of cationic trypsinogen within the pancreas, activating the digestive zymogenesis cascade and resulting in pancreatic autodigestion. The most common mutations of *PRSS1* are c.365G>A (p.R122H), c.364C>T (p.R122C), c.86A>T (p.N29I) and c.47C>T (p.A16V), and they have been proven to be causative for both hereditary CP and ICP [16]. Moreover, *SPINK1* can inhibit up to 20% of trypsin activity and may constitute one major mechanism to protect the pancreas from autodigestion [17]. The variants c.101A>G (p.N34S) and c.194+2T>C (IVS3+2T>C) are the predominant mutations of *SPINK1* that may contribute to the development of CP [18, 19].

The distribution of these identified mutations varies globally, including among Asian countries. While studies from Japan and China have shown that the c.194+2T>C variant of *SPINK1* may represent a unique genetic background for CP [20, 21], recent research from India reported a higher prevalence of the c.101A>G variant [22]. Furthermore, factors associated with pathogenic genetic variants in CP patients differ across studies [23, 24]. In Vietnam, data on CP and the role of genetic mutations in its development remain limited. In particular, the significance of *SPINK1* and *PRSS1* mutations in CP, their interactions with various etiological factors, and the risk factors related to these mutations are still poorly understood. Therefore, the present study aimed to identify *PRSS1* and *SPINK1* mutations and their associated factors in Vietnamese CP patients.

## 2 Methods

### 2.1 Study Participants

This cross‐sectional study was carried out from December 2022 to June 2024 at the Department of Gastroenterology, Cho Ray Hospital, Ho Chi Minh City, Vietnam. The study enrolled all consecutive patients over 18 years old who were diagnosed with CP according to the 2020 American College of Gastroenterology (ACG) Clinical Guidelines [25]. To ensure the homogeneity of the study population, focus on the most common causes, and minimize confounding factors, only patients with alcoholic CP (ACP), smoking‐associated CP (SCP), and ICP were recruited. The exclusion criteria included patients with any malignant diseases, those unwilling to participate in the research, and CP patients with other etiologies, such as hereditary CP, metabolic CP, traumatic CP, and autoimmune CP.

Data on demographics, clinical characteristics, etiologies, history of previous acute pancreatitis, morphologic features, gene mutations, and therapeutic interventions were recorded and evaluated. The cumulative amount of smoking (pack‐years) was equal to the number of packs of cigarettes smoked per day multiplied by the number of years of smoking. Alcohol intake was expressed as grams of alcohol consumed per day.

All eligible patients signed a written informed consent form. The study protocol was approved by the Board of Ethics in Biomedical Research of the University of Medicine and Pharmacy at Ho Chi Minh City (ID number: 756/HDDD‐DHYD, signed on 20/10/2022).

### 2.2 Definitions

The diagnosis of CP was based on the 2020 ACG Clinical Guidelines for CP [25]. Patients presenting with clinical symptoms suggesting inflammatory disorders of the pancreas (such as previous episodes of acute pancreatitis, characteristic pain, and/or maldigestion) would have cross‐sectional imaging for diagnostic confirmation. Computed tomography (CT) or magnetic resonance imaging is recommended as the first‐line test for CP diagnosis. In this study, contrast‐enhanced abdominal CT was the imaging test chosen for all patients. The major imaging features of CP on CT scan included the presence of parenchymal calcifications, marked pancreatic ductal changes (such as intraductal calcifications, dilatation or strictures of the pancreatic duct) or a combination of these findings [26, 27].

The etiology of CP can be classified into three categories: ACP, SCP, and ICP. ACP was defined as a history of alcohol intake of ≥ 80 g/day for males and 60 g/day for females for at least 2 years, irrespective of smoking status [28]. Patients with a smoking history of more than 30 pack‐years were assigned to the SCP [29]. Participants without identified causes of CP were categorized as having ICP.

Patients who met any of the following criteria were diagnosed with diabetes mellitus (DM) in accordance with the American Diabetes Association's guidelines: (1) a random venous plasma glucose concentration ≥ 11.1 mmol/L; (2) a fasting plasma glucose concentration ≥ 7.0 mmol/L; (3) HbA1c ≥ 6.5%; or (4) a history of DM with ongoing medication for hyperglycemia [30].

Complications of CP, including pseudocysts, bile duct obstruction, splenic vein thrombosis, pseudoaneurysm, intraductal papillary mucinous neoplasm, and pancreatic cancer, were diagnosed according to standard imaging criteria. Pancreatic leakage was diagnosed through evaluation of ascitic or pleural fluid, defined by the combination of a serum‐based gradient below 1.1 g/dL, a total protein level greater than 3 g/dL, and elevated ascitic amylase (> 1000 units/L) [31].

Genetic testing was defined as positive if known pathogenic genetic variants were present in at least one of the two genes, *PRSS1* or *SPINK1*. Patients with variants of unknown significance and no other pathogenic variants were categorized as having negative pathogenic variants.

### 2.3 Mutational Analysis

For each patient, 2 mL of peripheral blood was collected using EDTA as an anticoagulant. Genomic DNA was extracted via the GeneJET Genomic DNA Purification Kit (Thermo Scientific, USA) and stored at −30°C. PCR and sequencing primers were designed to analyze the sequences of exons and exon–intron boundaries of the *PRSS1* and *SPINK1* genes. The reference genomic sequences of the *PRSS1* and *SPINK1* genes were obtained from the National Center for Biotechnology Information Consensus CDS database, with accession numbers NG_008307.3 and NG_008356.2, respectively (https://www.ncbi.nlm.nih.gov/projects/CCDS/CcdsBrowse.cgi).

The *PRSS1* and *SPINK1* exons and exon‐intron boundaries were amplified with primers synthesized by IDT (Integrated DNA Technologies) and are listed in Table 1. The PCRs (15 μL) contained 25–50 ng of genomic DNA, 0.5 U of Taq Hot Start Polymerase (Takara Bio), 0.1 μM each forward and reverse primer, 200 μM each dNTP, and 1X PCR Buffer. The reactions were run in a SimpliAmp Thermal Cycler (Thermo Scientific) with the annealing temperature set to 58°C. The PCR products were analyzed via 1.5% agarose gel electrophoresis and then purified with ExoSAP‐IT reagent (Thermo Scientific). The amplicons were directly sequenced via the Sanger method via the BigdyeTM Terminator v3.1 Cycle Sequencing Kit (Applied Biosystems) in both the forward and reverse directions. The sequencing products were analyzed using an ABI 3500 Genetic Analyzer (Applied Biosystems).

**TABLE 1:** Primer sequences for PCR amplification.

Primer name	Primer sequence (5′–3′)	Length (bp)	Region
PRSS1‐1F	TGACCCTCACCTCACAGTCA	1404	Exons 1–2
PRSS1‐2R	CCAACCTCAGTAGTTCCCTG		
PRSS1‐3F	ATGAGCAGAGAGCTTGAGGA	1394	Exons 3–5
PRSS1‐5R	AGACAGTGAGAACAGGGTCA		
SPINK1‐1F	CCTTGCTGCCATCTGCCATA	262	Exon 1
SPINK1‐1R	CTCTCGAAGACTAGACTACA		
SPINK1‐2F	ACAGTCTGCAATGAAAGCAG	229	Exon 2
SPINK1‐2R	CTCCTCTTAACTTCAGGCTA		
SPINK1‐3F	ATCACAGTTATTCCCCAGAG	297	Exon 3
SPINK1‐3R	TTCTCGGGGTGAGATTCATA		
SPINK1‐4F	CCCCCTGTTTTTCTCCCATA	182	Exon 4
SPINK1‐4R	TCAACAATAAGGCCAGTCAG		

The sequencing results of the *PRSS1* and *SPINK1* genes were analyzed with CLC Mainworkbench v5.5 software based on the transcript versions NM_002769.5 and NM_003122.5, respectively, and nucleotides were counted from the first ATG translation initiation codon, calling mutants according to the T. den Dunnen nomenclature [32]. To determine the pathogenicity of the novel identified variants, functional prediction software packages and databases, including PolyPhen‐2, Clinvar, and Varsome, were used.

### 2.4 Statistical Analysis

Continuous variables are presented as the means ± standard deviations for normally distributed data or medians and interquartile ranges for skewed distributions. Categorical variables are expressed as frequencies and percentages. Non‐normally distributed continuous variables were compared between the two groups via the Whitney *U* test and across multiple groups via the Kruskal–Wallis rank sum test. Fisher's exact test was applied for categorical data. Univariate logistic regression was performed to estimate crude odds ratios (ORs) and their 95% confidence intervals (CIs) for the associations between independent and mutational variables. Variables with a *p* < 0.05 in the univariate analysis were included in the multivariate logistic regression model to adjust for potential confounders. Adjusted ORs with 95% CIs are reported. All *p*‐values less than 0.05 were considered statistically significant.

## 3 Results

### 3.1 Patient Characteristics

A total of 160 patients with CP were included in this study, comprising 100 individuals (62.5%) with ACP, 9 (5.6%) with SCP, and 51 (31.9%) with ICP. The median age of the patients was 50 years, ranging from 38 to 60 years. The majority of patients were male, accounting for 85.6% of the patients. The median BMI was 19.3 kg/m^2^ (16.9–21.4 kg/m^2^).

### 3.2 Distribution of Specific Genetic Variants According to the Etiology of CP

There were 64 patients (40%) with at least one pathogenic genetic variant in the *PRSS1* or *SPINK1* genes. Table 2 summarizes the frequency of *PRSS1* and *SPINK1* mutations in the ACP, SCP, and ICP groups.

**TABLE 2:** Distribution of specific genetic variants according to the etiology of chronic pancreatitis.

Gene	Variant	Total (*n* = 160)	ACP (*n* = 100)	SCP (*n* = 9)	ICP (*n* = 51)	*p*
*SPINK1*	c.101A>G (het)	33 (20.6%)	17 (17.0%)	1 (11.1%)	15 (29.0%)	0.2
	c.101A>G (hom)	5 (3.1%)	1 (1.0%)	0 (0%)	4 (7.8%)	0.085
	c.101A>G (total)	38 (23.7%)	18 (18.0%)	1 (11.1%)	19 (37.2%)	**0.025**
	c.194+2T>C (het)	22 (13.8%)	9 (9.0%)	1 (11.1%)	12 (23.5%)	0.049
	c.194+2T>C (hom)	1 (0.6%)	0 (0%)	0 (0%)	1 (2.0%)	0.4
	c.194+2T>C (total)	23 (14.3%)	9 (9.0%)	1 (11.1%)	13 (25.4%)	**0.024**
	c.206C>T (het)	5 (3.1%)	3 (3.0%)	1 (11.1%)	1 (1.9%)	0.3
	c.206C>T (hom)	1 (0.6%)	0 (0%)	0 (0%)	1 (1.9%)	0.4
	c.206C>T (total)	6 (3.8%)	3 (3.0%)	1 (11%)	2 (3.9%)	0.4
	c.101A>G (het)+c.194+2T>C (het)	6 (3.8%)	1 (1.0%)	0 (0%)	5 (9.8%)	**0.047**
	c.101A>G (het)+c.206C>T (hom)	1 (0.6%)	0 (0%)	0 (0%)	1 (1.9%)	0.4
	c.194+2T>C (het)+c.206C>T (het)	1 (0.6%)	0 (0%)	0 (0%)	1 (1.9%)	0.4
	Total	59 (36.8%)	29 (29.0%)	3 (33.3%)	27 (52.9%)	**0.012**
*PRSS1*	c.623G>C (het)	5 (3.1%)	4 (4.0%)	0 (0%)	1 (1.9%)	0.7
	c.410C>T (het)	1 (0.6%)	1 (1.0%)	0 (0%)	0 (0%)	0.9
	c.346C>T (het)	1 (0.6%)	1 (1.0%)	0 (0%)	0 (0%)	0.9
	Total	7 (4.4%)	6 (6.0%)	0 (0%)	1 (1.9%)	0.6
*SPINK1* + *PRSS1*	c.101A>G(het) c.410C>T(het)	1 (0.6%)	1 (1.0%)	0 (0%)	0 (0%)	0.9
	c.101A>G(het) c.623G>C(het)	1 (0.6%)	1 (1.0%)	0 (0%)	0 (0%)	0.9
Total		64 (40.0%)	33 (33.0%)	3 (33.3%)	28 (54.9%)	**0.034**

*Note:* The data are presented as *n* (%). Statistical test: Fisher's exact test. Bold indicates statistical significant value *p* < 0.05.

Abbreviations: ACP, alcoholic chronic pancreatitis; het, heterozygous; hom, homozygous; ICP, idiopathic chronic pancreatitis; SCP, smoking‐associated chronic pancreatitis.

### 3.3 Mutational Analysis of *SPINK1*

*SPINK1* gene variants were found in 59 of the 160 (36.8%) CP patients. Among these patients, 27 (52.9%) were ICP, which was significantly greater than the detection rate in ACP (29.0%) and SCP patients (33.3%) (*p* < 0.05).

A detailed analysis revealed that c.101A>G was the most common variant, present in 38 (23.7%) of 160 patients. Among them, 33 patients (20.6%) were in a heterozygous state, and 5 patients (3.1%) were in a homozygous state. c.194+2T>C was found in 23 patients (14.3%), 22 of whom (13,8%) were heterozygous, and 1 patient (0.6%) was homozygous. The distribution of these genetic variants differed significantly among the CP etiology groups (*p* < 0.05). Notably, 6 patients (3.8%) carried both heterozygous c.101A>G and heterozygous c.194+2T>C. In addition, c.206C>T was detected in 6 patients (3.8%), including 5 patients (3.1%) in a heterozygous state and 1 patient (0.6%) in a homozygous state. However, no significant differences were observed in the frequency of this variant among the ACP, SCP, and ICP groups (Table 2).

### 3.4 Mutational Analysis of *PRSS1*

The analysis of the *PRSS1* gene identified mutations in 7 of 160 CP individuals (4.4%), all of which were heterozygous. Among these, c.623G>C (p.G208A) was found in 5 patients (3.1%), c.410C>T (p.T137M) was found in 1 patient (0.6%), and c.346C>T (p.R116C) was present in 1 patient (0.6%). There was no significant difference in the frequency of *PRSS1* mutations across the various etiological groups of CP (*p* = 0.6) (Table 2).

### 3.5 Clinical Characteristics, Complications, and Morphologic Features of CP Patients With Genetic Mutations

We compared the clinical characteristics, complications, and morphological features of CP patients with and without genetic mutations, and the results are presented in Tables 3, 4, 5.

**TABLE 3:** Clinical characteristics of chronic pancreatitis patients with and without genetic mutations.

Clinical features	Mutation (*n* = 64)	No mutation (*n* = 96)	*p*
Age (years)	43 (33–55)	54 (42–61)	**< 0.001**
Male, *n* (%)	53 (82.8%)	84 (87.5%)	0.4
BMI (kg/m^2^)	19.4 (16.4–21.0)	19.5 (17.0–21.7)	0.4
Etiology			**0.034**
ACP	33 (51.6%)	67 (69.8%)	
SCP	3 (4.7%)	6 (6.3%)	
ICP	28 (43.8%)	23 (24.0%)	
Recurrent acute pancreatitis	47 (73.4%)	60 (62.5%)	0.15
Number of prior acute pancreatitis episodes	2.0 (0.0–5.0)	1.0 (0.0–3.3)	**0.021**
Abdominal pain	57 (89.1%)	83 (86.5%)	0.6
Diarrhea	6 (9.4%)	7 (7.3%)	0.6
Weight loss	42 (65.6%)	57 (59.4%)	0.4
Diabetes mellitus	37 (57.8%)	38 (39.6%)	**0.024**
Endoscopy intervention	10 (15.6%)	16 (16.7%)	0.9
Surgery intervention	20 (31.3%)	9 (9.4%)	**< 0.001**

*Note:* Data are presented as the median (25%–75%); *n* (%). Bold indicates statistical significant value *p* < 0.05.

Abbreviations: ACP, alcoholic chronic pancreatitis; BMI, body mass index; ICP, idiopathic chronic pancreatitis; SCP, smoking‐associated chronic pancreatitis.

**TABLE 4:** Complications among chronic pancreatitis patients with and without genetic mutations.

Complications	Mutation (*n* = 64)	No mutation (*n* = 96)	*p*
Biliary obstruction	12 (18.8%)	17 (17.7%)	0.9
Splenic vein thrombosis	7 (10.9%)	11 (11.5%)	0.9
Pseudoaneurysm	2 (3.1%)	3 (3.1%)	0.9
Pancreatic fistula	3 (4.7%)	3 (3.1%)	0.7
Intraductal papillary mucinous neoplasm	1 (1.6%)	7 (7.3%)	0.15
Pancreatic cancer	3 (4.7%)	3 (3.1%)	0.7

**TABLE 5:** Morphologic features of chronic pancreatitis patients with and without genetic mutations.

Imaging features	Mutation (*n* = 64)	No mutation (*n* = 96)	*p*
Pancreatic calcifications, *n* (%)	54 (84.4%)	82 (85.4%)	0.9
Pancreatic duct stones, *n* (%)	50 (78.1%)	50 (52.1%)	**< 0.001**
Pancreatic duct diameter (mm)	7.0 (5.0–9.3)	6.0 (4.0–9.0)	0.3
Pancreatic cysts, *n* (%)	7 (10.9%)	29 (30.2%)	**0.004**

*Note:* The data are presented as *n* (%). Statistical test: Fisher's exact test. Bold indicates statistical significant value *p* < 0.05.

According to the univariate analysis, genetic mutations were significantly related to age, etiology of CP, number of previous acute pancreatitis episodes, DM, and previous surgical intervention. Nevertheless, no significant differences were observed between CP‐related complications and genetic mutations (Tables 3 and 4). With respect to the morphologic features of CP, patients with mutations were significantly more likely to have pancreatic duct stones (78.1% vs. 52.1%) (*p* < 0.001). Conversely, pancreatic cysts were more prevalent in the mutation‐negative group (30.2% vs. 10.9%; *p* < 0.05). The other imaging features of CP did not differ significantly between these two groups (Table 5).

In the multivariate model, we found that age (OR: 0.95; 95% CI: 0.92–0.98; *p* < 0.05), DM (OR: 2.55; 95% CI: 1.11–6.04; *p* < 0.05), pancreatic duct stones (OR: 7.08; 95% CI: 2.81–20.40; *p* < 0.001) and surgical treatment (OR: 4.14; 95% CI: 1.34–14.10; *p* < 0.05) were independently associated with genetic mutations (Table 6).

**TABLE 6:** Logistic regression analysis for factors associated with gene mutations.

Risk factors	Univariable		Multivariable	
	OR (95% CI)	*p*	OR (95% CI)	*p*
Age (years)	0.96 (0.94–0.98)	< 0.001	0.95 (0.92–0.98)	**0.002**
Diabetes mellitus	2.09 (1.10–4.01)	0.024	2.55 (1.11–6.04)	**0.030**
Number of prior AP episodes	1.10 (1.02–1.19)	0.016	1.09 (0.99–1.22)	0.083
Etiology				
ACP				
SCP	1.02 (0.20–4.11)	0.9	—	—
ICP	2.47 (1.24–4.98)	0.010	2.37 (0.97–5.96)	0.061
Pancreatic duct stones	3.29 (1.64–6.90)	0.001	7.08 (2.81–20.40)	**< 0.001**
Pancreatic cysts	0.28 (0.11–0.66)	0.006	0.53 (0.17–1.51)	0.3
Surgery intervention	5.37 (2.28–13.8)	< 0.001	4.14 (1.34–14.10)	**0.017**

*Note:* Bold indicates statistical significant value *p* < 0.05.

Abbreviations: CI, confidence interval; OR, odds ratio.

## 4 Discussion

To the best of our knowledge, this is the first study evaluating the prevalence and distribution of *PRSS1* and *SPINK1* gene mutations, as well as their associated factors, among Vietnamese CP patients. Our findings revealed a high prevalence of the *SPINK1* gene variants c.101A>G and c.194+2T>C, particularly among patients with ICP, whereas *PRSS1* mutations were detected at a relatively low frequency. Moreover, we identified independent associations between these genetic mutations and specific clinical factors, including age, DM, pancreatic duct stones, and previous surgical intervention.

The overall prevalence of *SPINK1* mutations in CP patients is approximately 36.8%. Among them, the c.101A>G variant was the most prevalent mutation (23.7%), followed by the c.194+2T>C variant (14.3%). Both variants were significantly more common in the ICP group (52.9%) than in the ACP (29%) and SCP (33.3%) groups. Interestingly, the co‐occurrence of these mutations in some patients (3.8%) suggests potential synergistic effects that merit further investigation. We reported similar results in previous studies in India, in which the c.101A>G variant was present in up to 42%–44% of ICP patients and 17% of ACP patients [22]. Nevertheless, our findings contrast with reports from East Asian populations. A large study in China revealed a high prevalence of *SPINK1* mutations (nearly 38%) in the ICP group, which was significantly higher than the detection rates in ACP and SCP patients (*p* < 0.0001). Nonetheless, the most common variant in this study was c.194+2T>C, unlike the dominance of c.101A>G observed in our study [21]. Similarly, studies from Korea and Taiwan have shown the dominance of the c.194+2T>C mutation in ICP patients [33, 34]. Furthermore, the frequency of *SPINK1* mutations in non‐Asian ICP populations varies considerably, ranging from 15.8% in French patients [35], 19.6% in German patients [36], 28.6% in Polish patients [37], to as high as 37% in American patients [38], with the most common variant being c.101A>G across these studies. The discrepancy in the frequency and distribution of *SPINK1* mutations across populations may suggest a potential influence of ethnicity and geography on the genetic predisposition to CP. In addition, the higher frequency of *SPINK1* mutation in ICP than in ACP and SCP reflects the genetic basis of idiopathic forms of CP. These findings highlight the interplay between genetic and regional environmental factors, emphasizing the importance of population‐specific research to unravel the complex pathogenesis of CP.

*PRSS1* mutations are strongly associated with hereditary pancreatitis [39]. However, their roles in ICP and CP of other etiologies are still controversial. In our study, *PRSS1* mutations were identified in 7 patients (4.4%), including 1 patient with c.410C>T (p.T137M) (0.6%), 5 patients with c.623G>C (p.G208A) (3.1%) and 1 patient with c.346C>T (p.R116C) (0.6%). Among these, c.410C>T is classified as a benign variant, c.623G>C as predisposing, and c.346C>T as pathogenic, according to the Franco‐Chinese GREPAN Study Group [40]. In a recent study from China, *PRSS1* mutations were found in 7.55% of ICP patients, with the highest being c.365G>A (p.R122H) [24]. The results from a study conducted in India revealed that none of the 113 ICP patients had either the c.365G>A (p.R122H) or c.86A>T (p.N29I) variants of the *PRSS1* gene [22]. In a study from Germany, *PRSS1* variants were found in 8.3% of patients, and the common variants were c.365G>A (p.R122H) (3.8%) and c.47C>T (p.A16V) (2.1%) [36]. Similar findings were reported in a study conducted in France, which identified *PRSS1* mutations in 9.1% of patients with ICP, including the variants c.86A>T (p.N29I), c.365G>A (p.R122H), c.364C>T (p.R122C), and c.47C>T (p.A16V) [35]. The relatively low frequency of *PRSS1* mutations observed in our study is consistent with findings from other studies, emphasizing their limited role in nonhereditary CP.

Our findings revealed a significant inverse association between age and genetic mutations (OR: 0.95, 95% CI: 0.92–0.98, *p* < 0.05), indicating a greater prevalence of mutations in younger patients. This finding is consistent with reports from China, where younger age at diagnosis was significantly correlated with genetic mutations [24]. Similar results were reported in another study from Taiwan, which demonstrated that patients with *SPINK1* mutations had an earlier age of onset [34]. A large multicenter European cohort also demonstrated that patients carrying *SPINK1* mutations were significantly younger at diagnosis [41]. These findings reinforce the hypothesis that genetic factors play a dominant role in early‐onset CP, whereas environmental exposures, such as alcohol and smoking, accumulate over time to contribute to CP in older patients [25]. However, studies from India and Korea revealed no significant differences in the age of onset between ICP patients with and without mutations [22, 42]. Similarly, a study from the USA revealed that patients with *SPINK1* mutations had a younger age of onset, but the difference did not reach statistical significance [38]. Consequently, this crucial issue needs to be further investigated.

In our study, DM was independently associated with genetic mutations (OR: 2.55, 95% CI: 1.11–6.04; *p* < 0.05). This relationship is consistent with findings from a study among the Chinese population, where DM was more common in ICP patients with the *SPINK1* c.194+2T>C mutation (39.6%) than in those without the mutation (9.2%) (*p* < 0.001) [43]. Nonetheless, this finding remains controversial because several studies have reported no associations between DM and gene mutations. Sandhu et al. from America reported that the frequency of DM in ICP patients with the *SPINK1* c.101A>G mutation was greater than that in those without mutation, although the difference was not statistically significant [38]. A multicenter European cohort from France and Germany revealed no difference in DM prevalence between CP groups with and without *SPINK1* mutations [41]. Studies conducted in China also reported no associations between DM and *PRSS1* mutation in ICP patients. Similarly, another study in Poland revealed no significant differences in DM and *PRSS1* or *SPINK1* mutations among ACP and ICP patients [34, 37]. Therefore, further investigations should be carried out to clarify this issue.

Pancreatic duct stones, direct sequelae of CP, can occur in up to 50% of patients, with their prevalence increasing over time to 50% and 100% at 5 and 14 years after disease onset, respectively [44]. In our study, pancreatic duct stones showed the strongest association with genetic mutations (OR: 7.08, 95% CI: 2.81–20.40, *p* < 0.001). This finding is consistent with a study from China, which demonstrated that *SPINK1* mutations significantly increased the risk of pancreatic duct stones (OR, 11.07; *p* = 0.003) [45]. Another Chinese study also revealed that mutation‐positive patients had a greater prevalence of pancreatic stones (90.28% vs. 78.11%, *p* < 0.001) [24]. Similar results were reported in a Korean study, in which pancreatic duct stones occurred more frequently in patients with the *SPINK1* c.194+2T>C and *PRSS1* p.G208A pathogenic variants [42]. These findings emphasize the universal role of genetic predispositions in ductal stone formation, suggesting that genetic mutations may serve as valuable diagnostic and prognostic markers in mutation‐related CP.

Our results also revealed that prior surgical intervention was independently associated with genetic mutations (OR: 4.14, 95% CI: 1.34–14.10, *p* < 0.05). This aligns with findings from Japan, where patients with *SPINK1* c.101A>G mutations underwent surgical interventions more frequently [46]. A similar trend was also observed in another study from Japan in hereditary CP, which indicated that patients without the *PRSS1* or *SPINK1* mutations had significantly longer times to surgery [47]. These results suggest that genetic mutations may exacerbate disease progression and severity, necessitating more aggressive treatment approaches. However, contrasting findings from studies in China, Poland, France, and England have reported no associations between genetic mutations and surgical intervention [34, 37, 41]. As a result, additional studies should be conducted in the future to better investigate this association.

Our current study had several limitations. First, it was conducted at a single center in Ho Chi Minh City, potentially limiting the generalizability of the findings to the broader Vietnamese population. Second, the cross‐sectional design inherently restricts the ability to establish causal relationships between genetic mutations and the risk factors for CP. Third, we focused mainly on *PRSS1* and *SPINK1* mutations, and other important genetic factors implicated in CP, such as *CFTR, CTRC*, and *CEL*, were not investigated. This narrow focus may overlook the polygenic nature of the disease and its interaction with other potentially significant genetic mutations. Therefore, further extensive, longitudinal, and multigene studies are needed to gain a more comprehensive understanding of CP in the Vietnamese population.

## 5 Conclusion

In conclusion, this study provides the first comprehensive analysis of *PRSS1* and *SPINK1* mutations in Vietnamese CP patients. *SPINK1* mutations, particularly c.101A>G and c.194+2T>C, were highly prevalent, especially in ICP, whereas *PRSS1* mutations were less common. Independent factors associated with genetic mutations were younger age, DM, pancreatic duct stones, and prior surgical intervention. These findings underscore the genetic complexity of CP and highlight the need for population‐specific research to guide personalized diagnostic and therapeutic strategies in Vietnam.

## Ethics Statement

Ethical approval for this study was approved by the Board of Ethics in Biomedical Research of the University of Medicine and Pharmacy at Ho Chi Minh City (ID number: 756/HDDD‐DHYD, signed on 20/10/2022). All eligible patients signed a written informed consent form prior to study inclusion.

## Consent

All eligible patients signed a written informed consent form prior to study inclusion.

## Conflicts of Interest

The authors declare no conflicts of interest.
